# Understanding *Streptococcus suis *serotype 2 infection in pigs through a transcriptional approach

**DOI:** 10.1186/1471-2164-12-253

**Published:** 2011-05-20

**Authors:** Manli Liu, Liurong Fang, Chen Tan, Tiansi Long, Huanchun Chen, Shaobo Xiao

**Affiliations:** 1 Division of Animal Pathogens, State Key Laboratory of Agricultural Microbiology, Huazhong Agricultural University, Wuhan 430070, China; 2 College of Veterinary Medicine, Huazhong Agricultural University, Wuhan 430070, China

## Abstract

**Background:**

*Streptococcus suis *serotype 2 (*S. suis *2) is an important pathogen of pigs. *S suis 2 *infections have high mortality rates and are characterized by meningitis, septicemia and pneumonia. *S. suis *2 is also an emerging zoonotic agent and can infect humans that are exposed to pigs or their by-products. To increase our knowledge of the pathogenesis of meningitis, septicemia and pneumonia in pigs caused by *S. suis *2, we profiled the response of peripheral blood mononuclear cells **(**PBMC), brain and lung tissues to infection with *S. suis *2 strain SC19 using the Affymetrix Porcine Genome Array.

**Results:**

A total of 3,002 differentially expressed transcripts were identified in the three tissues, including 417 unique genes in brain, 210 in lung and 213 in PBMC. These genes showed differential expression (DE) patterns on analysis by visualization and integrated discovery (DAVID). The DE genes involved in the immune response included genes related to the inflammatory response (CD163), the innate immune response (TLR2, TLR4, MYD88, TIRAP), cell adhesion (CD34, SELE, SELL, SELP, ICAM-1, ICAM-2, VCAM-1), antigen processing and presentation (MHC protein complex) and angiogenesis (VEGF), together with genes encoding cytokines (interleukins). Five selected genes were validated by qRT-PCR analysis.

**Conclusions:**

We studied the response to infection with *S. suis *2 strain SC19 by microarray analysis. Our findings confirmed some genes identified in previous studies and discovered numerous additional genes that potentially function in *S. suis *2 infections in vivo. This new information will form the foundation of future investigations into the pathogenesis of *S. suis*.

## Background

*Streptococcus suis *(*S. suis *2) is an important pathogen of pigs that causes high mortality and is responsible for considerable economic loss to the porcine industry [1]. Serotype 2 is considered the most virulent form of the bacteria and is the serotype most frequently isolated from diseased animals [2]. *S. suis *2 is also an emerging zoonotic agent and has been isolated from a wide range of mammalian species, including humans, who are often infected via skin wounds during contact with pigs and their products [3]. *S. suis *2 is frequently isolated from asymptomatic pigs, especially adult pigs (young pigs are susceptible to the disease), which indicates that pigs can be carriers of *S. suis *2. Two outbreaks in humans have been documented in China, in 1999 and 2005; hundreds of people were infected and 52 died. Human illness following *S. suis *infection has also been reported in Thailand [4], the United Kingdom [5], the Netherlands [6], Australia [7] and the United States [8]. Moreover, in Hong Kong, infection with *S. suis *is the third most common cause of community-acquired bacterial meningitis, and in Vietnam it is the leading cause of meningitis in adult humans [9].

Previous studies of the pathogenesis of *S. suis *infection focused on the virulence factors of *S. suis *2. The capsular polysaccharide (CPS), muramidase-released protein (MRP), extracellular protein factor (EF) and suilysin (SLY) are considered to be virulence-associated factors that play an important role in the process of infection with *S. suis *2. In the Chinese strains, an 89 kb pathogenicity island (PAI) was found which has not been detected in other *S. suis *isolates [10].

Essentially, the disease is the result of the interaction between the bacteria and the host, and we believe further investigation of the host response to infection with *S. suis *2 will help us to better understand the disease. Several previous studies have shown that *S. suis *2 can adhere to various types of epithelial cell [11] and to microvascular endothelial cells from porcine brain [12,13]. In addition, the response of alveolar macrophages to *S. suis *2 infection is associated with induction of the nuclear factor-kappa B (NF-κB) and MAP-kinase signaling pathways. Further study has found that THP-1 cells respond to infection with *S. suis *2 by changes in the level of expression of TLR2 and CD14. The Toll-like receptors (TLRs) have been shown to recognize a wide range of microbial components and mediate responding cellular signaling. CD14 has been shown to be important in the recognition of LPS (lipopolysaccharide) [14]. The CD14 receptor is also known to interact with members of the TLR family. The receptors share common signaling pathways that trigger the association of adaptor molecules, such as myeloid differentiation factor 88 (MyD88), with several kinases, leading to the nuclear translocation of NF-κB. These findings indicate that these pattern recognition receptors may play a role in the host interaction with *S. suis *2. The purpose of our studies was to evaluate the interaction of the PBMC, brain and lung of pigs with *S. suis *2, and thereby facilitate further investigations on the pathogenesis of *S. suis *2.

## Results

### Transcription analysis

**Figure 1:**
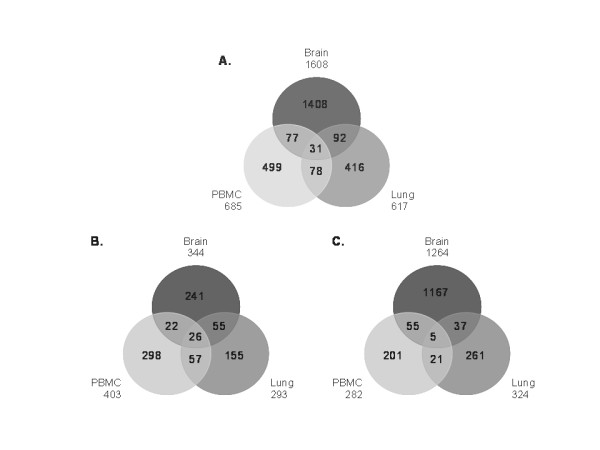
**Number of genes expressed differentially in brain, lung and PBMC**. The number of genes found to be differentially expressed in each of the tissues is shown in the circles. The grey circle represents the number of genes in the brain, tinted grey represents the PBMC and deep grey circle represents the lung. A. Number of DE genes including up-regulated and down-regulated. B. Number of up-regulated genes. C. Number of down-regulated genes.

In this study, differential gene expression was investigated among the three tissues studied (PBMC, brain and lung). Transcript analysis indicated that at least 48.19% and up to 67.89% of the probe sets were well detected in all 18 samples. The criteria of fold change (FC) ≥ 2 and p value ≤0.05 were considered to indicate up-regulation; FC ≤ 0.5 and p ≤ 0.05 were considered to represent down-regulation. Genes whose relative transcription levels were between 0.5 and 2 were considered to show no significant change. As shown in Figure 1, 1608 genes in the brain showed changes, including 344 genes up-regulated and 1264 genes down-regulated. There were 617 genes were changed in the lung, 293 up-regulated and 324 down-regulated and 685 genes were changed in PBMC, 403 up-regulated and 282 down-regulated. The expression of 31 genes was found to change in response to infection in all three tissues, comprising 26 genes up-regulated and 5 genes down-regulated.

All differentially expressed (DE) genes were annotated on the basis of the gene ontology (GO) database using Visualization and Integrated Discovery (DAVID). In brain tissue, of the 1608 DE transcripts, 460 were annotated by DAVID and these represented 417 unique genes. In the lung, 240 transcripts were annotated, which represented 210 unique genes of the 617 DE transcripts. In PBMC, of the 685 DE transcripts, 248 transcripts were annotated and represented 213 unique genes.

**Figure 2:**
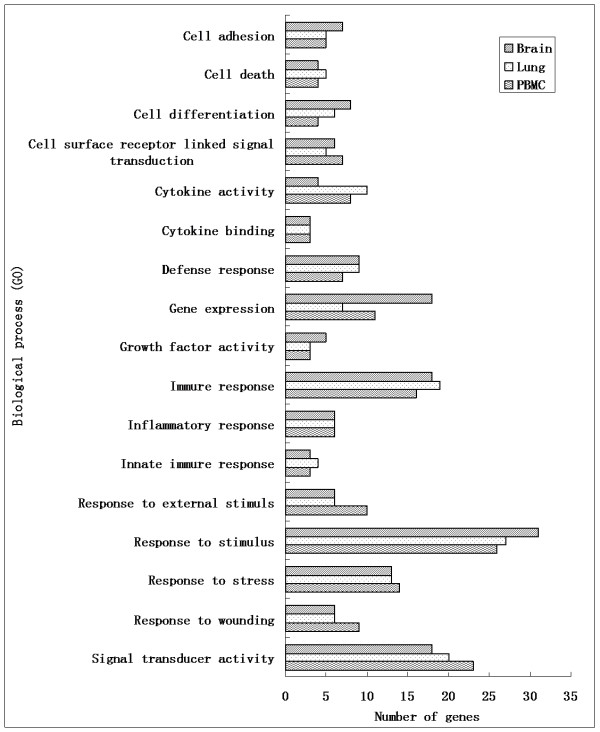
**Categories of genes divided on the basis of Gene Ontology (GO)**. Many categories shared the same transcripts

Figure 2 lists 17 categories of genes divided on the basis of gene ontology (GO), including those associated with the defense response, immune response, inflammatory response, innate immune response, cell adhesion, cell death, cell differentiation, cell surface receptor linked signal transduction, cytokine activity, cytokine binding, response to external stimuli, response to stimulus, response to stress, response to wounding, signal transducer activity, gene expression and growth factor activity.

### Quantitative RT-PCR to validate the microarray data

To validate the DE genes identified in the microarray data, quantitative RT-PCR (qRT-PCR) analysis was performed on the same samples that were used in the microarray. Three up-regulated genes and two down-regulated genes were selected for validation (Table 1). The three up-regulated genes analyzed were CD163 (cluster of differentiation 163), ICAM-1 (inter-cellular adhesion molecule 1) and TLR2; all three showed higher expression in infected groups tissues than in the control groups. Of the two down-regulated genes analyzed, VEGFA (vascular endothelial growth factor A) was found to be down-regulated in the lung and TTR (transthyretin) was found to be down-regulated in the brain and PBMC. Contrasting results were seen for VEGFA in the brain and PBMC and TTR in the lung. These contradictions between the qRT-PCR results and the microarray may arise from the qRT-PCR technique being more sensitive and accurate or that the primer design methods and the dynamic range were different.

**Table 1:** Verification of differentially expressed genes via qRT-PCR

Tissues	Genes	**Microarray FC**^**a**^	qRT-PCR FC	**p value**^**d**^
**Brain**	ICAM-1	+5.77	+^b ^6.00	0.0129
	TLR2	+3.86	+19.89	0.0301
	CD163	+8.91	+179.61	0.0158
	TTR	- ^c ^12.83	-10.92	0.0023
	VEGFA	+1.55	-4.20	0.0461
				
**Lung**	ICAM-1	+1.97	+55.50	0.0133
	TLR2	+2.44	+25.59	0.0216
	CD163	+4.25	+11.30	0.0056
	TTR	-1.05	-7.69	0.0292
	VEGFA	-2.22	-3.45	0.0201
				
**PBMC**	ICAM-1	+3.16	+13.13	0.0075
	TLR2	+2.70	+8.57	0.0132
	CD163	+9.08	+3.39	0.0243
	TTR	-1.07	-0.69	0.0055
	VEGFA	-2.71	-1.27	0.0332

^a^Fold Change. ^b^Represents up regulation. ^c^Represents down regulation. ^d^Significance level of differential expression for a particular gene via qRT-PCR.

### Induction of genes related to the innate immune response

Analysis of the expression of porcine genes in three tissues after infection with *S. suis *2 showed that a large set of DE genes is involved in the immune response. TLR signaling plays an essential role in the innate immune response. TLR2 (up-regulated between 2.4- and 3.9-fold in the three tissues) and TLR4 (up-regulated 2- to 4.3-fold in brain and PBMC but not lung) both belong to the TLR family and are expressed most abundantly in peripheral blood leukocytes. They mediate the host response to Gram-positive bacteria and play a fundamental role in pathogen recognition and activation of innate immunity. Another receptor for bacteria, CD14 (up-regulated 2- to 3-fold in the three tissues) recognizes LPS of Gram-negative bacteria and cooperates with TLR4 (which also recognizes LPS) via MYD88, leading to inflammatory responses. In addition, LY96 (MD-2) (up-regulated 2.3-fold in brain) plays a role in the recognition of LPS together with TLR2 or TLR4. Moreover, CD14 has also been found to recognize peptidoglycan (PGN) of Gram-positive bacteria and to be were associated with SS2 [15]. The signal transducer MYD88 (up-regulated 2- to 3.4-fold in brain and lung and 1.9-fold in PBMC) is an adapter for almost all TLR (except TLR3) signaling pathways, and acts via interferon regulatory factor 7 (IRF7, up-regulated approximately 2-fold in brain and lung), leading to activation of NF-kappa-B, cytokine secretion and the inflammatory response. CXCL9 (chemokine (C-X-C motif) ligand 9, otherwise known as MIG; up-regulated 18.8-fold in brain and 8.4-fold in lung) is thought to be induced by gamma interferon (IFN-γ) and binds to CXCR3, which activates the chemotactic functions of CXCL10 and CXCL11.

### Induction of genes associated with the inflammatory response

The inflammatory response is part of the initial defensive reaction against infection or injury caused by chemical or physical agents. Several DE genes in this study are involved in the inflammatory response, including CD163, AMCF-II, CCL2 and CCL4. The expression of hemoglobin scavenger receptor CD163 (M130) was up-regulated more than 4-fold in all three tissues. This has been identified as a receptor that might play a role in immunoregulation and may be a valuable diagnostic parameter for monitoring macrophage activation in inflammatory conditions. Another scavenger receptor, AMCF-II (alveolar macrophage-derived chemotactic factor-II, up-regulated 3.6-fold in lung), has chemotactic activity for porcine cells and can be induced by LPS. It belongs to the intercrine alpha (chemokine CxC) family; its new designation is CXCL5 and the gene symbol is SCYB5. Cytokine CCL2 (chemokine (C-C motif) ligand 2, up-regulated 12.6-fold in brain) is thought to bind to chemokine receptors CCR2 and CCR4. CCL4 (chemokine (C-C motif) ligand 4, up-regulated 2.41-fold in brain and 3.88-fold in lung) is a CC chemokine with specificity for CCR5 (up-regulated 4-fold in PBMC) receptors. CD1D (down-regulated 2.72-fold in PBMC and 2.49-fold in lung) encodes a divergent member of the CD1 family of transmembrane glycoproteins, which are structurally related to the major histocompatibility complex (MHC) proteins and form heterodimers with beta-2-microglobulin (B2M, down-regulated 3.54-fold in brain and 2.98-fold in lung).

### Genes associated with cell adhesion

Cell adhesion molecules (CAMs) perform various functions in fundamental cellular processes, including polarization, movement, proliferation and survival [16]. We discovered several DE genes that were related to cell adhesion during *S. suis *2 infection, including CD34, ICAM-1, ICAM-2, VCAM-1, SELE, SELL and SELP. CD34 (CD34 molecule, down-regulated 2.36-fold in lung) is a monomeric cell surface antigen that is expressed selectively on human hematopoietic progenitor cells. It plays a role in early hematopoiesis by mediating the attachment of stem cells to the bone marrow extracellular matrix or directly to stromal cells, and was found as a carbohydrate ligand for selectins. In addition, VCAM1 (CD106, vascular cell adhesion molecule, up-regulated 5.46-fold in PBMC) is a type I membrane protein that is important in cell-cell recognition, and mediates leukocyte-endothelial cell adhesion and signal transduction. ICAM proteins are type I transmembrane glycoproteins that bind to the leukocyte adhesion protein LFA-1. ICAM-1 (CD54, intercellular adhesion molecule-1, up-regulated 5.77-fold in brain and 3.43-fold in PBMC) is typically expressed on endothelial cells and cells of the immune system and binds to integrins of type CD11a/CD18, or CD11b/CD18. It alters shear stress stimulated up-regulation of endothelial VCAM-1 and ICAM-1 in a BMP-4 and TGF-beta1-dependent pathway. In addition, ICAM-2 (CD102, intercellular adhesion molecule-2, up-regulated 3.55-fold in brain) plays a role in the cellular interactions associated with xenograft rejection, by blocking LFA-1-dependent cell adhesion in lymphocyte recirculation, and mediates adhesive interactions that are important for antigen-specific immune responses and surveillance. Another CAM, CD18 (ITGB2, integrin, beta 2 (complement component 3 receptor 3 and 4 subunit, up-regulated 2.13-fold in brain), is a receptor for ICAM1, ICAM2, ICAM3 and ICAM4, and combines with ITGAM or ITGAX to form a leukocyte-specific integrin called macrophage receptor 1 (Mac-1), or inactivated-C3b (iC3b) receptor 3 (CR3).

The selectins are Ca^2+^-dependent CAMs that bind fucosylated carbohydrates. SELE (selectin E, CD62E antigen, up-regulated 4.82-fold in lung) encodes proteins that are found in cytokine-stimulated endothelial cells and is thought to mediate the adhesion of blood neutrophils to cytokine-activated endothelium through interaction with PSGL1/SELPLG. SELL (selectin L, CD62L antigen, up-regulated 3.90-fold in lung) is a cell surface adhesion protein which mediates the adherence of lymphocytes to endothelial cells of high endothelial venules in peripheral lymph nodes and promotes the initial tethering and rolling of leukocytes in endothelia. SELP (CD62P antigen, selectin P, up-regulated 2.69-fold in brain) encodes a 140 kDa protein that is stored in the alpha-granules of platelets and Weibel-Palade bodies of endothelial cells, and mediates the interaction of activated endothelial cells or platelets with leukocytes.

### Antigen processing and presentation

Antigen processing and presentation is performed by antigen presenting cells that express antigen (peptide or lipid) on their cell surface in association with an MHC protein complex. The MHC protein complex is a transmembrane protein complex composed of an MHC alpha chain and, in most cases, either an MHC class II beta chain or an invariant beta2-microglobin chain, with or without a bound peptide, lipid or polysaccharide antigen. Multiple MHC proteins were identified as DE genes after infection with *S. suis *2. SLA-1 (*Sus scrofa *MHC class I antigen 1, PD1, 3-fold down-regulated in brain) is involved in the presentation of foreign antigens to cells of the immune system. SLA-6 (*Sus scrofa *MHC class I antigen 6, 2-fold down-regulated in PBMC) is similar to the gene HLA-G of *Homo sapiens*. SLA-DRA (*Sus scrofa *MHC class II antigen DR-alpha, 3.7-fold up-regulated in brain) is similar to HLA-DRA, does not have polymorphisms in the peptide binding portion and acts as the sole alpha chain for DRB1, DRB3, DRB4 and DRB5. SLA-DRB1 (*Sus scrofa *MHC class II histocompatibility antigen SLA-DRB1, 2.8-fold up-regulated in brain, 3.8-fold down-regulated in PBMC and 4.5-fold down-regulated in lung) contains all the polymorphisms that specify the peptide binding and is present in all individuals. The protein encoded by SLA-DQA1 (*Sus scrofa *MHC class II histocompatibility antigen SLA-DQA, 3.2-fold up-regulated in brain), and SLA-DQB1 (*Sus scrofa *SLA-DQ beta1, 4-fold up-regulated in brain) are two proteins that are required to form the DQ heterodimer, similar to HLA-DQA1 and HLA-DQB1. SBAB-1044B7.2 (SLA-DMB, *Sus scrofa *MHC class II, DM beta, down-regulated 2.59-fold in PBMC) is similar to HLA-DMB and is located in intracellular vesicles, plays a critical role in catalyzing the release of class II-associated invariant chain peptide (CLIP) from newly synthesized MHC class II molecules and frees the peptide binding site for acquisition of antigenic peptides. In B cells, the interaction between HLA-DM and MHC class II molecules is regulated by HLA-DO. SLA-DOA (major histocompatibility complex, class II, DO alpha, HLA-DMA, SLA-DMA, SLA-DOA) was 3-fold up-regulated in brain. TAP1 (Transporter 1, ATP-binding cassette, sub-family B (MDR/TAP), up-regulated 4.24-fold in brain and 2.14-fold in lung) belongs to the ABC (ATP-binding cassette) transporter family, is involved in the transport of antigens from the cytoplasm to the endoplasmic reticulum for association with MHC class I molecules, and acts as a molecular scaffold for the final stage of MHC class I folding, namely the binding of peptide.

### Cytokine activity

Cytokines function in control of the survival, growth, differentiation and effector function of tissues and cells that participate in the immune and inflammatory responses. Interleukins are a group of cytokines that are produced by white blood cells (leukocytes) and a wide variety of somatic cells. Monocytes, macrophages and endothelial cells promote the development and differentiation of T, B and other hematopoietic cells, and play an important role in the immune system. The results of this study showed that IL1A was up-regulated 5.6-fold in PBMC. IL1B was up-regulated 7.6-fold in lung. IL6 was up-regulated 4.4-fold in lung. IL8 was up-regulated 4.6-fold in lung. IL12B was up-regulated 13.8-fold in PBMC and 3.4-fold in lung. IL15 was up-regulated 3-fold in brain. IL18 was up-regulated 2.7-fold in lung. IL10RB (interleukin-10 receptor subunit beta, up-regulated 2.1-fold in brain, 3.8-fold in PBMC and 3.5-fold in lung) belongs to the cytokine receptor family; coexpression with IL10RA proteins has been shown to be required for IL10-induced signal transduction. TNF-a (tumor necrosis factor-alpha, up-regulated 4.0-fold in PBMC and 2.9-fold in lung) is a cytokine secreted mainly by macrophages, which is involved in a wide spectrum of biological processes including cell proliferation, differentiation and apoptosis and has been implicated in a variety of diseases, including autoimmune diseases, insulin resistance, and cancer. CD40 (up-regulated 3.3-fold in brain and 3-fold in lung) is a member of the TNF-receptor superfamily and mediates a variety of immune and inflammatory responses. SR-PSOX (scavenger receptor for phosphatidylserine and oxidized low density lipoprotein (OxLDL), up-regulated 7.08-fold in PBMC) is a scavenger receptor on macrophages, binds to CXCR6/Bonzo and binds specifically to OxLDL, which suggests that it may induce a strong chemotactic response and induce calcium mobilization.

Furthermore, many cytokines are involved in signaling systems. STAT1 (signal transducer and activator of transcription 1, up-regulated 3.6-fold in brain and 2.4-fold in lung), STAT2 (up-regulated 2.09-fold in brain), STAT3 (up-regulated 2.36-fold in brain) and STAT5A (up-regulated about 2-fold in all three tissues) belong to the STAT family, which is usually activated by Janus kinase (JAK) and dysregulation. They regulate growth, survival and differentiation in cells. JAK2 (Janus kinase 2, up-regulated 2.2-fold in PBMC) is required for the response to IFN-γ. IKBKG (inhibitor of kappa light polypeptide gene enhancer in B-cells, kinase gamma, up-regulated 2-fold in brain), also known as NEMO and IKK-γ, is the regulatory subunit of the inhibitor of the IκB kinase (IKK) complex and interacts with IKK-alpha and IKK-beta to activate nuclear factor-kappa-B and other pathways. NFKBIA (nuclear factor of kappa light polypeptide gene enhancer in B-cells inhibitor, alpha, IκBα, up-regulated 3-fold in brain and 2.2-fold in lung) is one member of a family of cellular proteins that inhibit the NF-κB transcription factor. TGFB1 (transforming growth factor, beta 1, up-regulated 2.79-fold in brain) is a member of the transforming growth factor beta (TGFB) family of cytokines, which are multifunctional peptides that regulate proliferation, differentiation, adhesion, migration and other functions in many cell types. TBK1 (TANK-binding kinase 1, up-regulated 2-fold in PBMC) is similar to IKB kinases and can mediate NFKB activation, including the phosphorylation and activation of IRF3 and IRF7.

### Angiogenesis

Angiogenesis is a biological process of blood vessel formation in which new vessels emerge from the proliferation of pre-existing blood vessels. VEGF is a growth factor that induces endothelial cell proliferation, promotes cell migration, inhibits apoptosis and induces permeabilization of blood vessels. It was down-regulated 2.7-fold in PBMC and 2.2-fold in lung. Two other angiopoietic factors that were down-regulated in lung in our study were ANGPT1 and its receptor TEK (TIE2). ANGPT1 can activate TIE2 by inducing tyrosine phosphorylation, and participates in the endothelial developmental processes later than and distinct from VEGF. It plays a crucial role in mediating the interactions of endothelium and the surrounding matrix and mesenchyme, and also mediates blood vessel maturation/stability [17]. These results indicate that infection with *S. suis *2 may influence the maturation and stability of blood vessels.

## Discussion

*Streptococcus suis *infection is considered a major problem in the global pig industry and has caused mass mortality in pigs, particularly during the past 20 years [1]. Studies of the pathogenesis of this infection have generally used human macrophages, porcine choroid plexus epithelial cells (PCPEC) or porcine brain microvascular endothelial cells to investigate the host response to *S. suis *2 in vitro. Recently, Li et al. analyzed swine spleen infected with SS2 [18]. While these results strengthened our understanding of the pathogenetic mechanisms of the bacterium, the mechanism of the interaction between *S. suis *2 and the host remained unclear. In this study we investigated changes in gene expression in the brain, lung and PBMC of pigs in response to *S. suis *2 infection.

### Differential expression in all three tissues

Our analysis found 31 genes to be differentially expressed in infected and non-infected brain, lung and PBMC (Table 2). Most of these genes were associated with the innate immune system. In previous studies, CD163 has been discovered to be a cellular receptor for PRRSV in CD14 positive cells and to mediate viral entry and uncoating [19,20]. Other studies have revealed that CD163 can be a macrophage receptor for the binding of bacteria [21] and that it can promote bacteria-induced production of proinflammatory cytokines during infection [22,23]. These findings indicate that CD163 may have a function in microbial infection. A further study showed that CD163 was associated with *S. suis *2 infection [9]. However, the function of CD163 in this setting was unclear. In our study, up-regulated expression of CD163 was detected in all three infected tissues. Other studies have shown that up-regulation of CD163 can be induced by IL-10 and IL-6, and concomitantly influenced by multiple cytokines [24,25]. In addition, expression of CD163 increases along the pathway of macrophage differentiation [26,27]. Although IL-6 and IL-10 are known to be induced by TLR2, their up-regulation also affects CD163 up-regulation [28]. Human TLR2 can associate with CD14 and play an important role in SS2 infection [15]. Given that CD163, TLR2 and CD14 were all found to be up-regulated in our study, in combination with other cytokines (IL6, IL10, etc.), we demonstrate that these genes are involved in *S. suis *2 infections. The function of CD163 in *S. suis *2 infection invites further research.

**Table 2:** Thirty one genes are differentially expressed in all three tissues (brain, lung and PBMC)^a^

			Brain		PBMC		Lung	
GeneBank ID	Gene name	Gene symbol	**p-value**^**b**^	FC	p-value	FC	p-value	FC
NM_213857.1	TIMP metallopeptidase inhibitor 1	TIMP1	8.10E-05	3.65181	7.18E-03	2.66352	8.08E-05	5.85375
NM_213771.1	interleukin 10 receptor, beta	IL10RB	1.57E-02	2.11795	2.82E-04	3.85676	4.93E-04	3.5312
AF135122.1	signal transducer and activator of transcription 5a	STAT5A	1.89E-02	2.52188	3.35E-02	2.26822	4.90E-02	2.1119
NM_213761.1	toll-like receptor 2	TLR2	5.34E-05	3.8551	7.47E-04	2.69655	1.68E-03	2.43616
BE232628	Similar to acetyl-coenzyme A acyltransferase 2	LOC100155327	2.26E-02	-2.18647	3.55E-02	-2.03229	2.94E-02	-2.09592
CF362301	similar to syntaxin 11	LOC100152253	7.32E-04	5.02375	2.36E-04	6.38426	2.29E-02	2.5498
BX917516	Similar to Histone H2A type 1-C (H2A/l)	LOC100155325	3.90E-02	2.42467	5.21E-04	6.01862	6.21E-05	9.92759
U55068.1	solute carrier family 11 (proton-coupled divalent metal ion transporters), membe	SLC11A1	4.73E-03	2.51987	1.33E-03	3.03397	1.01E-03	3.1645
NM_214107.1	adrenomedullin	ADM	3.01E-02	2.77106	2.67E-02	2.84834	8.15E-03	3.71455
NM_214127.1	superoxide dismutase 2, mitochondrial	SOD2	5.53E-03	7.15851	1.43E-03	11.0196	2.19E-03	9.60376
BP161394	CD14 molecule	CD14	3.31E-03	2.03944	3.19E-03	2.04785	1.00E-04	3.0381
NM_213976.1	CD163 molecule	CD163	3.28E-07	8.90964	2.99E-07	9.0756	2.28E-05	4.25237
AJ663261	solute carrier family 2 (facilitated glucose transporter), member 3	SLC2A3	8.45E-05	4.66825	2.26E-02	2.00129	2.66E-03	2.72289
CF368229	similar to Transcription factor ETV7 (Transcription factor Tel-2) (ETS-related p	LOC100156210	2.05E-02	2.21213	8.86E-03	2.53106	2.92E-02	2.08944
NM_001005351.1	interferon stimulated exonuclease gene 20kDa	ISG20	3.11E-03	4.68553	1.48E-02	3.28837	1.54E-03	5.51054
BI402345	---	---	4.97E-04	3.711	1.09E-03	3.27418	2.44E-03	2.88867
BQ603979	---	---	5.78E-04	3.34979	3.55E-04	3.61029	8.69E-03	2.26328
CF180654	---	---	1.18E-03	2.99848	2.09E-03	2.76134	1.13E-03	3.01962
CK457954	---	---	1.06E-06	-11.761	1.33E-03	-3.10658	1.56E-03	-3.03402
BF079328	---	---	1.84E-03	2.25759	2.51E-03	2.17989	7.8E-04	2.49339
BI402402	---	---	4.92E-07	14.747	1.82E-06	10.7931	1.17E-02	2.27742
CO946796	---	---	3.94E-06	-7.83105	4.95E-03	-2.42882	7.06E-03	-2.31103
BX924325	---	---	6.55E-03	2.41774	1.08E-02	2.24747	9.32E-03	2.29727
BX926199	---	---	4.86E-02	2.23094	1.14E-02	2.97827	8.5E-04	5.01781
CO953941	---	---	4.74E-02	4.00916	9.42E-04	15.4413	5.59E-03	8.31723
BQ597849	---	---	3.00E-04	-2.5804	2.70E-03	-2.03617	1.12E-03	-2.23346
BF443326	---	---	2.75E-03	4.19593	1.18E-02	3.10382	6.91E-03	3.46603
CK457453	---	---	1.95E-02	2.07169	2.39E-02	2.01081	1.77E-02	2.101
CN159527	---	---	9.44E-03	2.61444	1.02E-02	2.57932	2.39E-04	4.98165
CO954523	---	---	7.41E-03	-2.17839	8.56E-03	-2.13788	4.56E-03	-2.32115
BE232824	---	---	4.42E-06	2.8712	9.60E-05	2.15173	8.74E-05	2.16904

^a^Up/down-regulated genes which annotation based on GO term. ^b^Significance level of differential expression for a particular gene via microarray.

### Genes differentially expressed in PBMC

Infection with *S. suis *2 in both pigs and humans can cause fatal septicemia. Sepsis is a serious condition with a significant morbidity and mortality, and has been defined as a combination of the presence of infection and the systemic inflammatory response syndrome (SIRS) [29]. *S. suis *2 produces suilysin, which can result in hemolysis of red blood cells and the release of hemoglobin, and is toxic for epithelial cells [30,31], which may allow invasion of the bloodstream. Free *S. suis *2 cells or those in close contact with monocytes in the bloodstream may cause septicemia.

Cytokines, including TNF-α, IL-1, IL-10 and IL-12, have been shown to play an important role in septic shock [31-34], and were found in the present study to be significantly differentially expressed in PBMC after infection with *S. suis *2. Moreover, several scavenger receptor genes were differentially expressed in PBMC after infection with *S. suis *2, when compared with brain and lung. SR-PSOX (CXCL16) and Scarb2 were up-regulated only in PBMC. CXCR6, up-regulated in both lung and PBMC, is a ligand for CXCL16. CXCL16, and its ligand CXCR6, are involved in interactions of DC with T cells and in the regulation of T cell migration in the red pulp of the spleen; they are up-regulated by inflammatory mediators [35]. CXCL16 is a biomarker of inflammatory bowel disease and diseases such as sarcoidosis and atherosclerosis. It is possible that the scavenger receptor may play an important role in the effects of *S. suis *2 infection, especially septicemia. Another gene, VEGF, is a growth factor that is active in angiogenesis and endothelial cell growth. In our study, this gene was down-regulated, which suggests that *S. suis *2 infection may suppress vasculogenesis, endothelial cell proliferation and cell migration, and may enhance the production of septicemia by inhibition of VEGF expression,

### Genes differentially expressed in brain

Meningitis is another important clinical manifestation of *S. suis *2 infection. After *S. suis *2 bacteria enter the bloodstream, they cause an overwhelming sepsis that enables *S. suis *2 to pass between or through the cells that form the blood-brain barrier (BBB), allowing *S. suis *2 to disseminate in the central nervous system (CNS).

In our results, several genes involved in antigen processing and presentation [36] showed differential expression in porcine brain after *S. suis *2 infection, but were unchanged in PBMC and lung tissues. These genes belonged mainly to the class of swine leukocyte antigens (SLA), which is also called the swine major histocompatibility complex (MHC). Recent research has demonstrated that SLA molecules play an important role in porcine immune responses to pathogens and vaccines [37].

Class I molecules, such as SLA-1, are found on almost all cells and present proteins to cytotoxic T cells. They are thought to be recognized by xenoantigens [37,38] and have been implicated in human xenotransplantation [39] and cancer diseases such as porcine melanoma [40]. Class II molecules are found on certain immune cells, chiefly antigen-presenting cells (APCs) such as macrophages and B cells. They recognize non-self molecules, present the fragments to helper T cells, and stimulate an immune reaction. Class II molecules include SLA-DRA, SLA-DRB1, SLA-DQA1, SLA-DQB1, SLA-DMB and SLA-DOA. SLA Class II also has a function in CSFV (classical swine fever virus) [41], PRRSV (porcine reproductive and respiratory syndrome virus ) [42], and FMDV (foot and mouth disease) [43] infections in pigs. In addition, cytokines such as TNF-α, IFN-γ and IL-4 have been shown to regulate the expression of MHC class I and II antigens on transplanted organs that are involved in immune responses [44]. Human leukocyte antigens (HLA) have been proven to be associated with many diseases, and can inhibit lysis of NK cells in HIV infection by interacting with their inhibitory receptors [45]. Therefore, the up-regulated expression of cytokines and swine MHC molecules (SLA) may play an important role during *S. suis *2 infection.

### Genes differentially expressed in lung

Pneumonia is another important clinical manifestation of *S. suis *2 infection. *S. suis *2 can adhere to many cells in vitro, including several types of epithelial cell, porcine brain microvascular endothelial cells and alveolar macrophages [12,13,30]. Before it breaks through the epithelial cell barrier to enter the bloodstream, *S. suis *2 naturally colonizes the upper respiratory tract of pigs, particularly the tonsils and nasal cavity [46]. It is transmitted via the respiratory route by breaching the mucosal epithelium and then enters the blood vessels [2]. The capsule of *S. suis *2 and suilysin secreted by *S. suis *2 may help the bacterium to evade macrophage phagocytosis and innate immune responses [47,48]. In a previous study, *S. suis *2 was shown to interact with alveolar macrophages and induce the NF-kB and MAP-kinase signaling pathways. In this study, we evaluated the differential expression of genes in the lung in response to *S. suis *2 infection. According to our results, a group of cytokines showed maximal up-regulation in the lung after infection with *S. suis *2. Of these, IL1B, IL-6, IL-8 and IL18 were up-regulated only in lung, IL12B and TNF-a were more highly expressed in both lung and PBMC, and IL10RB in all three tissues. In addition, IL1A was up-regulated in PBMC while IL15 was up-regulated in brain but not in lung. These data are in agreement with other studies that suggest that *S. suis *2 can stimulate porcine monocytes or macrophages to produce proinflammatory cytokines, and thus contribute to the pneumonia caused by *S. suis *2 infection. In addition, we have shown that MHC molecules and adhesion molecules are critical in allowing *S. suis *2 to break through epithelial cell barriers to cause pneumonia, although many of the DE genes involved are little known in *S. suis *2 infection.

## Conclusions

Global transcription studies have been performed to analyze the pathogenesis of many diseases. In this study, we profiled the response of PBMC, brain and lung tissues to infection with *S. suis *2 strain SC19 using the Affymetrix Porcine Genome Array. A total of 3,002 differentially expressed transcripts were identified in the three tissues, including 417 unique genes in brain, 210 in lung, 213 in PBMC and 31 in all three tissues. These DE genes involve in the immune response included genes related to the inflammatory response, innate immune response, cell adhesion, antigen processing and presentation, cytokines and angiogenesis. In addition, a number of DE genes identified in our study are still function unknown, providing information for future investigations. Taken together, the transcriptome analysis of *S. suis *2-infected tissues will increase our knowledge of the pathogenesis of meningitis, septicemia and pneumonia in pigs caused by *S. suis *2.

## Methods

### Bacterial strains and growth conditions

*S. suis *2 strain SC19 was isolated from diseased pigs in the Sichuan region of China in 2005. Bacteria were grown on tryptic soy agar plates (DifcoTM) containing 10% bovine blood overnight at 37°C, and inoculated into tryptic soy broth (DifcoTM) containing 10% bovine blood for 6 h at 37°C. For experimental infection, the serum broth culture was centrifuged at 3,300 × g for 5 min to pellet the bacteria, washed twice with phosphate-buffered saline (PBS), and resuspended in PBS without serum to the appropriate dilution before infection.

### Animals and tissue collection

All animal tissues were collected in compliance with local animal experimentation legislation and with approval of the Biological Studies Animal Care and Use Committee of Hubei Province (Y20100566). At 40 days of age, six piglets free of *S. suis *2 were allocated randomly to the infected group and the uninfected group. Each piglet in the infected group was injected intravenously with *S. suis *2 strain SC19 at a dose of 3×10^5 ^colony-forming units (CFU). Each piglet in the uninfected group was treated similarly with an identical volume of PBS as control. At 24 h after challenge, the pigs were slaughtered and their brains, lungs and venous blood were collected with RNase-free equipment for microarray analysis. The brains and lungs from both groups were frozen immediately in liquid nitrogen. Venous blood were collected with anticoagulant citrate concentrated solution 4% (S5770, Sigma-Aldrich, St Louis, MO, USA) at a ratio of 9:1, and Histopaque (1077, Sigma-Aldrich) was used for isolation of PBMC according to the manufacturer's instructions. Finally, the PBMC were resuspended with isotonic PBS, and counted under a microscope. TRIzol reagent (Invitrogen) was added at a suitable volume and cells were stored at -80°C until used.

### RNA extraction and reverse transcription

Total RNA extraction from tissues was performed according to the standard instructions for TRIzol reagent (Invitrogen, Carlsbad, CA, USA) and clean-up was carried out using an RNeasy Mini Kit (Qiagen, Valencia, CA, USA). The integrity and concentration of RNA were evaluated using an Agilent 2100 Bioanalyzer (Santa Clara, CA, USA). Reverse transcription of the total RNA and synthesis of biotin-labeled cRNA with one round of amplification was carried out following the standard Affymetrix one-cycle protocol according to the manufacturer's instructions.

For qRT-PCR analysis, the tissues were ground to a powder with liquid nitrogen and an RNAprep pure Tissue Kit (DP431, TIANGEN, Beijing, China) was used for RNA extraction and purification, following the manufacturer's instructions. The PBMC were prepared with TRIzol reagent according to the TRIzol standard instructions (Invitrogen). The RNA was quantified by spectrophotometry (ND-1000; NanoDrop Technologies). The total RNA was reverse transcribed to cDNA using a first strand cDNA synthesis kit (ReverTra Ace -a-TM, TOYOBO) according to the manufacturer's instructions.

### Microarray hybridization

The RNA was labeled and hybridization was performed using the recommended protocols supplied by the manufacturer (Affymetrix, Santa Clara, CA, USA), by GeneTech Biotechnology Limited Company (Shanghai, China). Briefly, a sample of RNA was converted to double-stranded cDNA using a one-cycle cDNA Synthesis Kit (Affymetrix, Inc., Santa Clara, CA) and a T7-oligo (dT) primer. Synthesis of biotin-labeled cRNA from cDNA by in vitro transcription (IVI) was conducted using a GeneChip IVT Labeling Kit (Affymetrix). The cDNA and cRNA were purified using the Sample Cleanup Module (Affymetrix), quantified by spectrophotometric methods and checked by formaldehyde denaturing gel electrophoresis in 1.2% agarose gels. The samples were characterized by dispersed bands (28S and 18S) without any obvious smearing patterns from degradation. Subsequently, labeled cRNA was fractionated and hybridized with the GeneChip Porcine Genome Array according to the standard procedures provided by the manufacturer. The chips were washed and stained with a GeneChip Fluidics Station 450 (Affymetrix) using the standard fluidics protocol. The probe arrays were scanned using the Affymetrix^® ^GeneChip^® ^Scanner 3000.

### Microarray data analysis

For analysis of the microarray data, the Affymetrix expression data were analyzed using the software package Gene Spring, version 7.31 (Agilent). All experiments were performed in triplicate to allow the assessment of within-group variation. First, the expression data obtained from infected cells were normalized to the median of the uninfected control samples. An initial selection of genes was made based on a 2-fold change in expression relative to that of uninfected control cells. Subsequently, an error model based on replicate measurements was defined in Gene Spring, and the significance of differential expression was established using analysis of variance and the false-discovery rate method of Benjamini and Hochberg [49] to correct for multiple testing. Microarray analysis was then performed by GeneTech Company using an Agilent gene chip array. The data discussed in this study have been deposited in the NCBI Gene Expression Omnibus with accession number GSE24889.

### Quantitative RT-PCR (qRT-PCR) analysis

QRT-PCR was used to validate selected data from the microarray experiments and to follow the expression of a subset of genes over time. Five genes with different expression patterns (up-regulated, down-regulated and no significant change) were randomly selected. These genes were ICAM-1, TLR2, CD16, TTR and VEGFA. GAPDH (glyceraldehyde-3-phosphate dehydrogenase) was used as a endogenous control, designed using Primer Express 2.0 software (Applied Biosystems Inc., Foster City, CA, USA). The specific primers used for the various qRT-PCR assays are listed in Table 3. The SYBR Green method was used for qRT-PCR amplification. All reactions were performed in triplicate. Each reaction tube contained 25 μL 2 × SYBR Green real-time PCR Master Mix (TOYOBO QPK-201), 0.5 UmL^-1 ^RNAse Inhibitor, 0.3 UmL^-1 ^ReverTraAce, 0.4 mM gene-specific F and R primers and 5 μL template, made up to a final volume of 50 μL with distilled water. The qRT-PCR were performed in an ABI 7500 real-time PCR system (Applied Biosystems, Warrington, UK) as follows: 42°C for 30 min and 95°C for 5 min, followed by 40 cycles of 95°C for 15 s, 60°C for 30 s, and 72°C for 45 s. Melting curves were obtained from 55°C to 95°C, read every 0.5 °C for 10s, than cooling at 25°C for 30s. The melting curves were analyzed to prevent genomic DNA amplification, and the PCR products were confirmed by agarose gel electrophoresis (1.5%). Gene expression level was calculated using the comparative Ct method [50]. The duplicates for each reaction were averaged. Fold changes value were calculated 2^-ΔΔCt ^which ΔΔCt = (Ct_test gene_-Ct_test __GAPDH _) - (Ct_control gene_-Ct_control __GAPDH _). *t *tests were performed to calculate statistical significance of genes expression between the test group and control group.

**Table 3:** Primers for qRT-PCR

Gene name	Primer sequence (5'→ 3')	GenBank ID
CD163	Forward: TCAGACACTATCCCCGTGCA Reverse: GGCGAAGTTGACCACTCCC	NM_213976
ICAM-1	Forward: ACCCACACCTTGCTACCCCT Reverse: GTGGACCGTAGCCTCTGAGG	NM_213816
TLR2	Forward: CCATCTTCGTGCTTTCCGAG Reverse: GGCGGTGTCATCGTTCTCAT	NM_213761
TTR	Forward: TCGCTGGACTGGTGTTTGTG Reverse: GGACTGCCTCGGACAGCAT	NM_214212
VEGFA	Forward: GAGACCAGAAACCCCACGAA Reverse: GGCAGTAGCTGCGCTGGTA	NM_214084
GAPDH^a^	Forward: ACATGGCCTCCAAGGAGTAAGA Reverse: GATCGAGTTGGGGCTGTGACT	AF017079

^a^GAPDH was used as a endogenous control.

## Abbreviations

SS2: *Streptococcus suis *2; DE: differentially expressed; FC: fold change; GO: Gene Onotlogy; qRT-PCR: quantitative RT-PCR;

## Authors' contributions

ML and SX designed and performed the experiments. LF and CT made bioinformatics and statistical analysis. TL participated in the animal challenge experiment. The manuscript was written by ML, SX, and HC. All authors read and approved the final manuscript.
